# A randomised controlled trial of acceptance and commitment therapy for improving quality of life in people with muscle diseases

**DOI:** 10.1017/S0033291722000083

**Published:** 2023-06

**Authors:** Michael Rose, Christopher D. Graham, Nicola O'Connell, Chiara Vari, Victoria Edwards, Emma Taylor, Lance M. McCracken, Aleksander Radunovic, Wojtek Rakowicz, Sam Norton, Trudie Chalder

**Affiliations:** 1 Department of Neurology, King's College Hospital, Denmark Hill, Brixton, London, SE5 9RS, UK; 2 School of Psychology, Queen's University Belfast, David Keir Building, 18-30 Malone Road, Belfast BT9 5BN, Northern Ireland; 3 Department of Psychological Medicine, Institute of Psychiatry, Psychology and Neuroscience, King's College London, 16 De Crespigny Park, London, SE5 8AF, UK; 4 Department of Psychology, Uppsala University, Postal Box 1225, 751 42 Uppsala, Sweden; 5 Barts and the London MND Centre, Royal London Hospital, Whitechapel, London, EH1 1BB, UK; 6 Wessex Neurological Service, University Hospital Southampton, Tremona Road, Southampton SO16 6YD, UK; 7 Department of Psychology, Institute of Psychiatry, Psychology and Neuroscience, King's College London, 16 De Crespigny Park, London, SE5 8AF, UK; 8 Department of Inflammation Biology, Faculty of Life Sciences and Medicine, Centre for Rheumatic Disease, King's College London, Weston Education Centre, London, SE5 8AF, UK

**Keywords:** Muscle diseases, acceptance and commitment therapy, quality of life, randomised controlled trial

## Abstract

Abstract

**Background:**

Chronic muscle diseases (MD) are progressive and cause wasting and weakness in muscles and are associated with reduced quality of life (QoL). The ACTMuS trial examined whether Acceptance and Commitment Therapy (ACT) as an adjunct to usual care improved QoL for such patients as compared to usual care alone.

**Methods:**

This two-arm, randomised, multicentre, parallel design recruited 155 patients with MD (Hospital and Depression Scale ⩾ 8 for depression or ⩾ 8 for anxiety and Montreal Cognitive Assessment ⩾ 21/30). Participants were randomised, using random block sizes, to one of two groups: standard medical care (SMC) (*n* = 78) or to ACT in addition to SMC (*n* = 77), and were followed up to 9 weeks. The primary outcome was QoL, assessed by the Individualised Neuromuscular Quality of Life Questionnaire (INQoL), the average of five subscales, at 9-weeks. Trial registration was NCT02810028.

**Results:**

138 people (89.0%) were followed up at 9-weeks. At all three time points, the adjusted group difference favoured the intervention group and was significant with moderate to large effect sizes. Secondary outcomes (mood, functional impairment, aspects of psychological flexibility) also showed significant differences between groups at week 9.

**Conclusions:**

ACT in addition to usual care was effective in improving QoL and other psychological and social outcomes in patients with MD. A 6 month follow up will determine the extent to which gains are maintained.

## Introduction

Adult muscle diseases (MD), such as facioscapulohumeral, Becker and limb-girdle muscular dystrophy, and inclusion body myositis, principally affect muscle tissue causing weakness and insidious declines in mobility and other physical functioning, alongside pain, fatigue and other symptoms (Merrison & Hanna, 2009). There are no curative treatments for most MD and symptom burden can be high, presenting barriers to participation in activities of daily living, which can affect quality of life (QoL). In addition to symptom severity, many other factors influence QoL in MD (Graham, Rose, Grunfeld, Kyle, & Weinman, 2011). For example, mood is a consistent predictor of QoL (Graham et al., 2011), as is sleep (Kalkman, Schillings, Zwarts, van Engelen, & Bleijenberg, 2007) and employment status (Minis et al., 2010; Oksuz, Kilinc, & Yildirim, 2009). Given the range of contributing factors, there is considerable individual variation in QoL, such that even those with a high symptom burden may report high QoL.

Several psychological factors, such as beliefs about illness, coping methods (Graham et al., 2014) and psychological flexibility (Graham, Gouick, Ferreira, & Gillanders, 2016a), also explain some of the variation in QoL among people with MD (Graham et al., 2011). Consequently, interventions targeting these factors could offer additional means to retain or improve QoL (Graham, Simmons, Stuart, & Rose, 2015). Among candidate psychological interventions targeting the aforementioned psychological factors that are commonly used in clinical practice for long-term health conditions (Thewes et al., 2014) we selected Acceptance and Commitment Therapy (ACT) (Hayes, Luoma, Bond, Masuda, & Lillis, 2006). ACT is often argued to be particularly suited to improving QoL in the context of chronic diseases (Hadlandsmyth, White, Nesin, & Greco, 2013; Low et al., 2012). One key reason is that the model assumes that negative thoughts, feelings and emotions can be understandable responses to overcoming adversity, such as living well with a chronic condition. Therefore, instead of focusing therapy on controlling or reducing these naturally occurring experiences, which may offer limited scope for improvement, ACT aims to improve meaningful functioning, even with uncomfortable experiences present, by promoting psychological flexibility (Hayes et al., 2006). Psychological flexibility can be defined as: ‘…the capacity to persist or to change behaviour in a way that (1) includes conscious and open contact with thoughts and feelings (openness), (2) appreciates what the situation affords (awareness), and (3) serves one's goals and values (engagement)’ (McCracken & Morley, 2014, p. 225).

Therapy methods are then arranged to build skills in psychological flexibility, including techniques such as mindfulness practice, values-elicitation, goal-setting and perspective-taking (Graham, Gouick, Krahé, & Gillanders, 2016b; Hayes, 2019). Evidence suggests that ACT is effective for improving outcomes in chronic pain and possibly in mental health conditions (Gloster, Walder, Levin, Twohig, & Karekla, 2020; Hann & McCracken, 2014). While there have been a number of trials of ACT in chronic diseases, such as diabetes, epilepsy, and HIV, evidencing promising effects on outcomes like distress and well-being, these have largely been of a preliminary nature (Graham et al., 2016b). Powered, quality trials are required to draw stronger inference about the efficacy of ACT in this context.

To date, there have been two randomised controlled trials of psychological therapy in MD. Both have used a traditional cognitive behaviour therapy approach focused on improving fatigue and delivered to the sub-group of people with facioscapulohumeral MD (Voet et al., 2014) or myotonic dystrophy (Okkersen et al., 2018) reporting very severe fatigue. Assessment of the efficacy of psychological interventions targeting overarching outcomes that are commonly the focus of psychological therapy in clinical practice - such as QoL or mood - or with broader MD populations, is lacking.

Given the aforementioned clinical and theoretical applicability of ACT, we devised a home-based ACT intervention (Graham et al., 2017) for improving QoL and distress that we hoped would suit the mobility limitations caused by MD. We previously piloted the approach in a small case series, which suggested that this intervention could have utility (Graham et al., 2017; Rose et al., 2018). Therefore, in the present paper, we report on a subsequent multi-centred, two-armed, randomised controlled trial to evaluate the efficacy of our largely self-guided ACT plus standard medical care (SMC) compared with SMC alone, for improving QoL in those with MD.

## Methods

This study had a two-arm, randomised, multicentre, parallel design comparing a guided self-help ACT intervention plus ‘SMC’ with ‘SMC’ alone (Rose et al., 2018).

Ethical approval was received by the London-Camberwell St Giles Research Ethics Committee, UK (16/LO/0609). The trial was registered with ClinicalTrials.gov Identifier: NCT02810028, and a detailed published protocol is available (Rose et al., 2018).

Participants were recruited from three NHS MD clinics, the charity MD-UK and MD registers. Inclusion criteria were: adults (aged 18 years and older), diagnosed with one of four specific MDs, for more than six months, on the basis of the following diagnostic criteria: (1) Limb-girdle muscular dystrophy; symptomatic LGMD genetically or pathologically proven; (2) Dystrophinophathies resulting in a Becker Muscular Dystrophy phenotype with pathology or genetic diagnosis (excluding Duchenne muscular dystrophy as it is significantly life-limiting with neuro-cognitive involvement. The focus of this study was MDs with similar characteristics likely to result in sufficiently similar psychosocial challenges); (3) Facioscapulohumeral muscular dystrophy (FSH) diagnosed clinically with specific genetic abnormality in the subject or their family; or (4) Inclusion body myositis clinico-pathologically defined, clinically defined or probable IBM based on European Neuromuscular Centre (ENMC) research diagnostic criteria 2013 (Rose, 2013). Potential participants needed access to the internet and a computer to access intervention materials and have scores of ⩾ 8 for depression or ⩾8 for anxiety on the Hospital Anxiety and Depression Scale (HADS).

Potential participants were excluded if they had unstable complications of MD including neuromuscular respiratory weakness or cardiomyopathy, major active comorbidities unrelated to MD (such as arthritis, respiratory disease, cardiovascular disease), a current diagnosis of an active major mental health disorder likely to interfere with participation, current or recent participation in other treatment intervention studies (<4 weeks after completion), currently receiving psychological support or psychotherapy, inability to read English questionnaires, and cognitive impairment that would prevent comprehension of ACT modules and questionnaires (as assessed by the Montreal Cognitive Assessment 5 min protocol).

Participants were randomised to one of two groups: (1) SMC or (2) the ACT intervention in addition to SMC. Randomisation was conducted by an independent randomisation service at the King's Clinical Trial Unit. Randomisation was conducted at the level of the individual, using block randomisation with randomly varying block sizes stratified by recruiting site.

Blinding of patients and the trial therapist was not possible. The trial's research assistants responsible for the collection and inputting of data and the trial statistician were blinded to group allocation as were all other members of the research team, including the principal investigator.

The intervention was a guided self-help ACT intervention tailored to the experiences of those with MD. Acceptability was previously assessed in a case series (Graham et al., 2017), and the intervention is described in detail in the trial protocol (Rose et al., 2018). It consisted of four modules and corresponding audio files, supported by five 15–30 min telephone support sessions. Modules and audio files took between 45 and 90 min to complete and included common written and audio exercises designed to enhance psychological flexibility (Harris, 2009; Hayes, 2005). The modules and audio files were presented to participants as a series of four psychological skills to try out in their everyday life in order to enhance well-being. Skill 1, Mindfulness, included the practice of brief centring and willingness exercises; Skill 2, Unhooking, involved practising with methods for stepping back from entanglement with thoughts, including the practice of defusion/verbal distancing exercises; Skill 3, Follow your values, invited reflections on personal values, and encouraged the identification and practice of behaviours consistent with these values; Skill 4, Take an observer perspective, encouraged skills in flexible perspective-taking. Follow-up, telephone calls were delivered by a clinical psychologist. These calls gave participants an opportunity to discuss the modules, their experiences completing exercises and to reflect on whether or not and in which situations any new skills had been helpful in their everyday life.

The ACT therapist attended 3 days of training in the intervention prior to the initiation of recruitment. The therapist attended monthly supervision meetings with a clinical supervisor to ensure adherence to the trial protocol.

### Therapy integrity

Guided by Perepletchikova, Hilt, Chereji, & Kazdin's (2009) checklist for the assessment of treatment integrity, we used the ACT Fidelity Measure (ACT-FM) (O'Neill, Latchford, McCracken, & Graham, 2019) to assess fidelity to ACT. Scores on this measure ranged from 0–36, with higher scores indicating greater adherence to ACT principles. In addition, we used an ACTMuS-specific rating scale to assess the trial therapist's competence and fidelity to the ACTMuS intervention as well as overall therapeutic alliance (possible scores 0–7). All telephone therapy sessions were audio-recorded. Following completion of the study, two experienced ACT clinicians, independent to the research team, rated all of session 2 (*n* = 65) and 34 of session 3 therapy sessions. Coders were trained over three meetings, which involved listening to and rating several treatment sessions together.

#### Standard medical care (SMC)

All participants received SMC throughout the trial in line with current medical practice. This consisted of a review of functional impairment arising from muscle weakness and suggestions to help reduce associated disability with home adaptations and assistive devices, the monitoring of respiratory and cardiac complications, recommendations for local physiotherapy input, and answering any queries on the condition, often using information leaflets from MD-UK or a disease support group.

Participants were followed up at 3, 6, and 9 weeks after randomisation. Baseline measures were conducted face-to-face in muscle clinics or over the telephone. Participants completed follow-up measures online using the Bristol Online Survey (now called Online Surveys; https://www.onlinesurveys.ac.uk/) delivered via an email link.

### Outcome measures

The primary outcome was QoL as assessed by the Individualised Neuromuscular Quality of Life Questionnaire (INQoL) – life area domains, which is the average of five subscales measuring the impact of MD on activities, independence, social functioning, emotional functioning, and body image (Vincent, Carr, Walburn, Scott, & Rose, 2007). The INQoL has been demonstrated to be reliable and valid, with Cronbach's estimates showing good internal consistency scores of greater than 0.70 across each life area domain (Sansone et al., 2010).

Secondary outcomes included patient-reported total scores on the weakness, fatigue and pain subscales of the INQoL, the Work and Social Adjustment Scale (WSAS) (Mundt, Marks, Shear, & Greist, 2002), Hospital Anxiety and Depression Scale (HADS) (Zigmond & Snaith, 1983), the Stanford Health Assessment Questionnaire Disability Index (HAQ-DI) (Fries, Spitz, & Young, 1982), Acceptance and Action Questionnaire (AAQ-II) (Bond et al., 2011), Mindfulness Attention Awareness Scale (MAAS) (Brown & Ryan, 2004), Committed Action Scale (CAQ) (McCracken, Chilcot, & Norton, 2015), IBM Functional Rating Scale (Jackson, Barohn, Gronseth, Pandya, & Herbelin, 2008), Patient Global Impression of Change scale (PGIC) (Ferguson & Scheman, 2009), and patients' rating of treatment satisfaction measured on a scale of 1–7 from ‘very dissatisfied’ to ‘very satisfied’.

Initially, to help characterise our sample, we included physiotherapy assessment of MD severity with the Adult Ambulatory Neuromuscular Assessment (ANA) (Mayhew et al., 2011) score, the 6-minute walk test (6MWT), and with Manual Muscle Strength Testing (MMST) (Cuthbert & Goodheart, 2007) assessing the strength of 12 different muscles bilaterally on the MRC 6 point scale giving a total score of 0 to 120, with higher scores representing levels of strength closest to normal levels. However, the requirement for participants to attend neuromuscular centres for these physiotherapy assessments was significantly slowing recruitment and these assessments were dropped. Hence baseline measures of MMST, 6MWT, and ANA are missing for some participants.

The original sample size calculation indicated that 154 participants were needed, with 77 participants in each arm, to detect a standardised mean difference of 0.5 (medium magnitude) in the primary outcome based on a 2-sided 5% significance and 20% loss to follow-up at 9 weeks. This corresponds to a difference between 11.5 and 15.8 points on each of the INQoL domains. No information is currently available regarding clinically important differences for the INQoL, however an effect size of this magnitude typically relates to a clinically important difference for health-related QoL instruments (Norman, Sloan, & Wyrwich, 2003).

Analysis and reporting of this trial were in accordance with Consolidated Standards of Reporting Trials (CONSORT) guidelines (Altman et al., 2001). Statistical analyses were conducted according to the Statistical Analysis Plan version 1.1 dated July 2017 by a statistician blind to treatment group allocation. In brief, estimates of treatment effect at the 3, 6 and 9-week post-randomisation assessments were based on adjusted mean differences using linear-mixed models following the intention-to-treat principle. A two-level model was estimated including a random intercept to account for repeated assessments within individuals over time. Covariates in the model included an indicator variable for group assignment, an indicator for follow up time, an interaction term for a group by time, the baseline level of the outcome variable, and indicator variables for the centre as this was a stratification factor in the randomisation. Robust standard errors were estimated using the Huber-White sandwich estimator to counter the biasing effect of deviations from non-normality of the residuals on estimated standard errors, and thus *p* values and confidence intervals.

Planned sensitivity analysis involved consideration of the impact of missing follow up data and protocol deviations using the following methods, respectively: (i) imputing missing follow up data using a baseline observation carried forward approach; (ii) missing not at the random pattern-mixture model (i.e. relaxing the missing at random assumption for the primary outcome); and (iii) per-protocol analysis for all outcomes excluding those not receiving treatment or deviating substantially from the protocol. Further sensitivity analysis considered the impact of deviations in the timing of the follow-up assessment from the scheduled dates of completion using time in weeks as a continuous variable. This made a negligible difference to the treatment effect estimates and is not presented here. There were no planned subgroup analyses.

Analyses were conducted using Stata MP 15.1 with the analysis reproducible by saved statistical code.

## Results

**Fig. 1.**
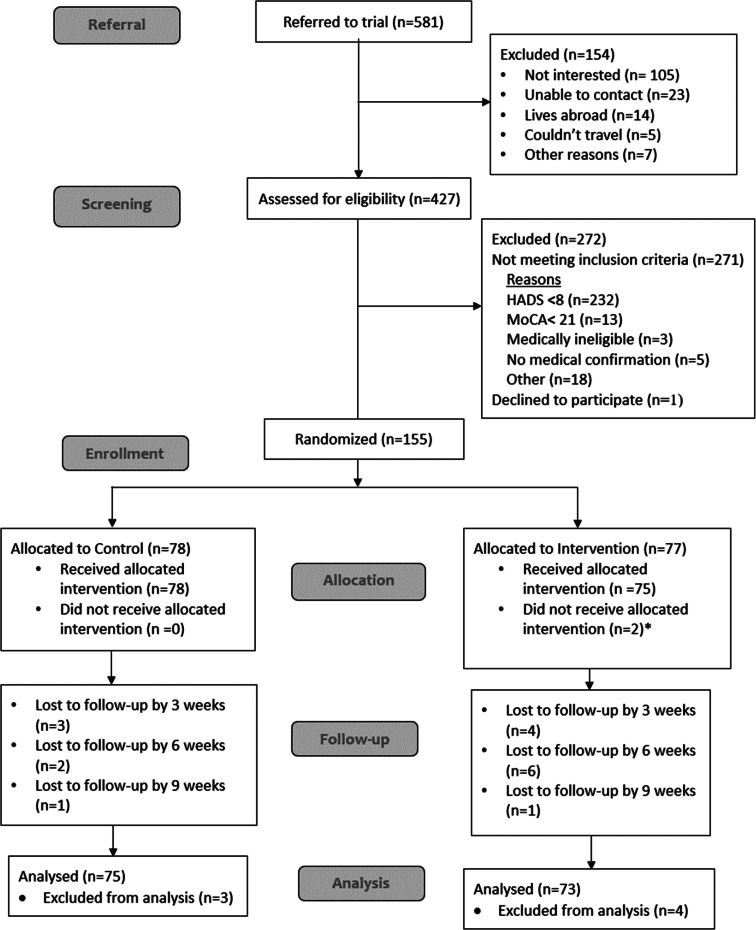
Consort diagram. * Five total withdrawals, of whom 2 received one session, two received 2 sessions and 1 received three sessions – in CONSORT have listed the two receiving only one session as ‘did not receive allocated intervention’).

A summary CONSORT diagram is presented in Fig. 1. Between July 2016 and April 2018, 155 of 427 patients screened were eligible and consented to participate in the trial.

Thus, 155 participants were randomised, exceeding the target sample size by one. Seventy-seven participants were randomised to the intervention arm and 78 to standard care. Loss to follow up was lower than expected, with 138 (89.0%) completing the full 9-weeks of follow-up. In total, 148 (95.5%) patients providing data on at least one follow-up occasion were retained for the intention-to-treat analysis, irrespective of whether they received treatment.

53 (68.8%) patients in the treatment group received all five ACT telephone sessions, 11 (14.3%) received four ACT sessions, five (6.5%) received three ACT sessions, five patients received two ACT sessions (6.5%) and three (3.9%) received only one ACT session. The average duration of session one was 16.77 min (s.d.: 2.3), session two was 30.3 min (s.d.: 3.8), session three was 28.2 min (s.d.: 5.8), session four was 27.3 min (s.d.: 6.8) and session five was 16.3 min (s.d.: 3.4).

Five participants withdrew from treatment in the intervention arm but not from the trial as a whole. Of these, two patients had completed one session, two patients had completed two sessions, and one patient had completed three sessions before the withdrawal.

The demographics of the sample are in Table 1. The demographics of the sample - including years with MD and disability level - was comparable to a recent sample recruited from national health service clinics (Graham et al., 2014). Table 2 gives the baseline values for the objective measures (AANA, MMST, 6MWT) of muscle function for those (*n* = 89) who attended a centre for these physiotherapy-conducted measures. Those randomised to the intervention (*n* = 48) had comparable muscle function to those randomised to SMC (*n* = 41) and participants were a mixture of both ambulant and non-ambulant.

**Table 1.** Categorical baseline variables by treatment group

	Control (*n* = 75)		Intervention (*n* = 73)		Total	
	*n*	%	*n*	%	*N*	%
Gender						
Female	31	41.3	42	57.5	73	49.3
Male	44	58.7	31	42.5	75	50.7
Total	75	100.0	73	100.0	148	100.0
MD type						
Limb Girdle	16	21.3	19	26.0	35	23.6
Beckers	2	2.7	2	2.7	4	2.7
FSHD	36	48.0	38	52.1	74	50.0
IBM	21	28.0	14	19.2	35	23.6
Total	75	100.0	73	100.0	148	100.0
Other physical health diagnoses						
No	30	41.1	36	49.3	66	45.2
Yes	43	58.9	37	50.7	80	54.8
Total	73	100.0	73	100.0	146	100.0
Ethnicity						
White British	64	85.3	61	83.6	125	84.5
White Irish	2	2.7	0	0.0	2	1.4
Other White	2	2.7	4	5.5	6	4.1
Asian Indian	3	4.0	3	4.1	6	4.1
Asian Pakistani	0	0.0	2	2.7	2	1.4
Other Asian	1	1.3	0	0.0	1	0.7
Black Caribbean	1	1.3	1	1.4	2	1.4
Black African	1	1.3	1	1.4	2	1.4
Other	1	1.3	1	1.4	2	1.4
Total	75	100.0	73	100.0	148	100.0
Occupational status						
Employed (full-time)	18	24.0	23	31.5	41	27.7
Employed (part-time)	10	13.3	12	16.4	22	14.9
Unemployed	10	13.3	10	13.7	20	13.5
Student	1	1.3	1	1.4	2	1.4
Retired	31	41.3	22	30.1	53	35.8
Voluntary Work	0	0.0	1	1.4	1	0.7
Other	5	6.7	4	5.5	9	6.1
Total	75	100.0	73	100.0	148	100.0
Education						
No formal education	5	6.7	1	1.4	6	4.1
GCSE/0 Level or equivalent	26	34.7	23	31.5	49	33.1
A level or equivalent	12	16.0	17	23.3	29	19.6
Degree	16	21.3	19	26.0	35	23.6
Postgraduate	9	12.0	8	11.0	17	11.5
Other	7	9.3	5	6.8	12	8.1
Total	75	100.0	73	100.0	148	100.0
Marital status						
Single	16	21.3	17	23.3	33	22.3
Married	36	48.0	43	58.9	79	53.4
Living with partner	11	14.7	7	9.6	18	12.2
Separated	0	0.0	2	2.7	2	1.4
Divorced	8	10.7	4	5.5	12	8.1
Widowed	3	4.0	0	0.0	3	2.0
Other	1	1.3	0	0.0	1	0.7
Total	75	100.0	73	100.0	148	100.0
Dependents						
No	53	70.7	45	61.6	98	66.2
Yes	22	29.3	28	38.4	50	33.8
Total	75	100.0	73	100.0	148	100.0
Possible anxiety disorder						
No	23	30.7	20	27.4	43	29.1
Yes	52	69.3	53	72.6	105	70.9
Total	75	100.0	73	100.0	148	100.0
Possible depressive disorder						
No	24	32.0	19	26.0	43	29.1
Yes	51	68.0	54	74.0	105	70.9
Total	75	100.0	73	100.0	148	100.0
ACT experience						
No	73	97.3	73	100.0	146	98.6
Yes	2	2.7	0	0.0	2	1.4
Total	75	100.0	73	100.0	148	100.0
Mindfulness experience						
No	56	74.7	55	75.3	111	75.0
Yes	19	25.3	18	24.7	37	25.0
Total	75	100.0	73	100.0	148	100.0
Other psychological experience						
No	42	56.0	47	65.3	89	60.5
Yes	33	44.0	25	34.7	58	39.5
Total	75	100.0	72	100.0	147	100.0
Treated depression						
No	43	57.3	45	61.6	88	59.5
Yes	32	42.7	28	38.4	60	40.5
Total	75	100.0	73	100.0	148	100.0
Treated anxiety						
No	60	80.0	49	67.1	109	73.6
Yes	15	20.0	24	32.9	39	26.4
Total	75	100.0	73	100.0	148	100.0
Part of patient group						
No	52	69.3	50	68.5	102	68.9
Yes	23	30.7	23	31.5	46	31.1
Total	75	100.0	73	100.0	148	100.0

**Table 2.** Continuous baseline variables by treatment group

	Control (*n* = 75)			Intervention (*n* = 73)			Total		
	*N*	Mean	s.d.	*N*	Mean	s.d.	*N*	Mean	s.d.
Age	75	54.5	15.3	73	50.8	15.3	148	52.7	15.4
Years since diagnosis	72	19.0	17.4	72	16.3	14.7	144	17.7	16.1
Age of onset	73	35.9	21.2	72	32.3	19.4	145	34.1	20.3
INQoL total	75	58.7	16.9	73	60.9	17.9	148	59.8	17.4
WSAS function	75	38.2	15.4	73	35.5	15.6	148	36.9	15.5
HADS anxiety	75	9.5	3.4	73	9.9	3.9	148	9.7	3.7
HADS depression	75	8.9	3.1	73	10.0	3.1	148	9.5	3.1
HAQ disability	75	1.8	0.8	72	1.7	0.7	147	1.8	0.8
MMST	41	90.5	22.3	48	92.1	19.8	89	91.4	20.8
ANA	41	15.9	11.9	48	16.0	10.6	89	16.0	11.2
6MWT	22	225.1	177.8	21	279.6	202.3	43	251.7	189.9

In total, 17 people were lost to follow-up during the course of the study (11 ACTMUS; 6 SMC). Of those, 9 explicitly withdrew or were withdrawn from the study (5 by 3-weeks; 3 by 6-weeks; 1 by 9-weeks) and 8 stopped providing data (2 by 3-weeks; 5 by 6-weeks; 1 by 9-weeks). Reasons for withdrawal mainly involved an unwillingness to complete questionnaires. In addition, one person withdrew from therapy but continued to provide data. In total, seven patients provided no post-randomisation follow up data, and were thus excluded from the main analyses. Those lost to follow-up tended to have lower educational attainment (Fisher's exact *p* = 0.034) and were less likely to have had prior experience of any kind of psychological intervention (Fisher's Exact *p* = 0.017). This is unlikely to have any biasing effect on the treatment effect analysis since the number of people lost to follow up was small, excluding <5% patients.

**Fig. 2.**
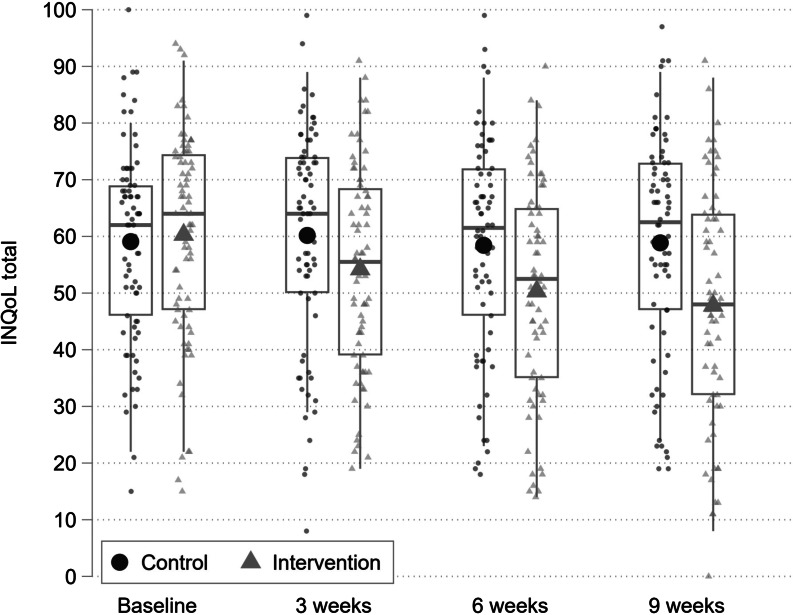
Boxplots describing the distribution of the INQoL total score (primary outcome) by group at each time point. Small markers indicate individual data points and large markers sample means.

Figure 2 shows boxplots describing the distribution of the INQoL total score (primary outcome) by group at each time point. Circles indicate individual data points and diamonds sample means.

The INQoL life area domains total score is scaled to range between 0 and 100 where higher scores indicate worse QoL. There is a clear trend for the INQoL life area domains total score to reduce over the three post-randomisation assessments for the treatment group, with no change observed for the control group. Table 3 indicates that the adjusted group difference favoured the treatment group and was significant with moderate to large effect sizes at all three-time points. Sensitivity analyses, using the per-protocol sample, that excluded 8 individuals who withdrew and were therefore assumed not to have received a sufficient dose of the treatment, indicated no substantive difference in the treatment effect estimate. Specifically, the standardised effects size at 9 weeks was increased from an SMD of −0.71 to −0.75.

**Table 3.** Treatment effects on primary outcome by analysis sample

		Control			Intervention			Adjusted mean difference			
Analysis sample	Time	*N*	Mean	s.d.	*N*	Mean	s.d.	Diff	s.e.	*p*	SMD
ITT	Baseline	75	58.69	16.94	73	60.92	17.91				
	3 weeks	74	60.18	19.13	72	54.19	18.29	−7.81	1.64	0.000	−0.45
	6 weeks	70	58.43	19.16	66	50.30	19.37	−9.36	1.90	0.000	−0.54
	9 weeks	72	58.86	19.57	66	47.77	21.18	−12.22	2.17	0.000	−0.71
LOCF	Baseline	78	59.08	16.94	77	60.29	18.04	–	–	–	–
	3 weeks	78	60.55	18.90	77	54.42	18.66	−7.44	1.58	0.000	−0.43
	6 weeks	78	58.54	19.60	77	51.17	19.75	−8.69	1.79	0.000	−0.50
	9 weeks	78	58.65	20.10	77	48.90	21.29	−11.11	2.02	0.000	−0.64
Per-protocol	Baseline	75	58.69	16.94	69	60.72	18.25	–	–	–	–
	3 weeks	74	60.18	19.13	68	54.31	18.52	−7.49	1.66	0.000	−0.44
	6 weeks	70	58.43	19.16	63	50.19	19.61	−9.43	1.98	0.000	−0.55
	9 weeks	72	58.86	19.57	63	47.08	21.23	−12.89	2.22	0.000	−0.75

Further sensitivity analysis examined the impact of missing follow up data on the treatment effect estimate at 9 weeks. Imputing missing data using the baseline observation carried forward approach indicated a negligible difference in the interpretation of the treatment effect. Specifically, the standardised effects size at 9 weeks was reduced from an SMD of −0.71 to −0.64. The robustness of the treatment effect was further supported using a pattern-mixture modelling approach, which indicated that the group differences would remain significant under any plausible missing data mechanism (see online Supplementary Material).

**Fig. 3.**
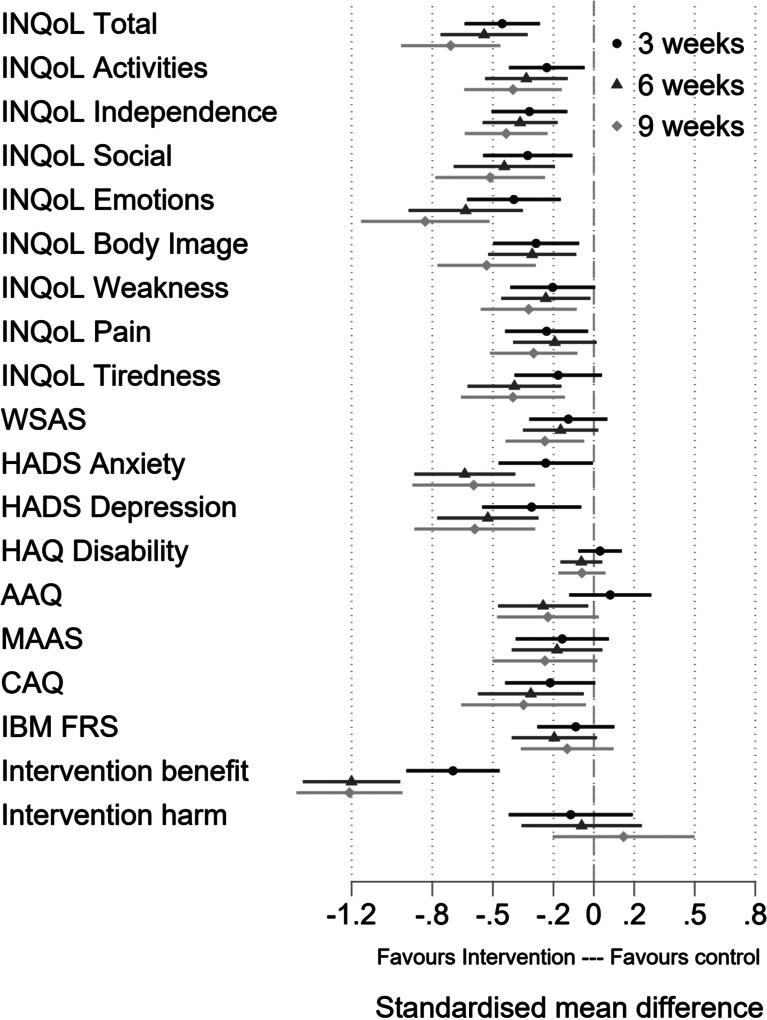
Forest plot of standardised effect sizes.

Figure 3 and Table 4 show the treatment effects for the secondary outcomes at each assessment based on the intention to treat the sample. Differences favour the treatment group with most being statistically significant at the 5% level at all three follow up assessments. Effect sizes are typically moderate to large. Only differences for the HAQ and MAAS are non-significant with small effects at all assessments.

**Table 4.** Treatment effects for the secondary outcomes by analysis sample

		Control			Intervention			Adjusted mean difference			
Variable	Time	*N*	Mean	s.d.	*N*	Mean	s.d.	Diff	s.e.	*p*	SMD
INQoL Activities	0	75	70.63	21.41	73	68.74	21.37				
	3	74	69.54	21.69	73	63.33	21.47	−4.90	2.01	0.015	−0.23
	6	71	68.66	23.20	65	59.66	21.64	−7.03	2.20	0.001	−0.33
	9	72	68.89	23.95	66	58.30	23.12	−8.43	2.60	0.001	−0.40
INQoL Independence	0	75	59.93	23.77	73	61.71	24.10				
	3	74	61.72	24.59	73	56.15	24.59	−7.49	2.25	0.001	−0.32
	6	71	62.10	22.98	66	54.58	22.24	−8.57	2.23	0.000	−0.37
	9	72	62.71	23.87	66	53.21	24.37	−10.20	2.46	0.000	−0.43
INQoL Social Relationships	0	72	41.11	20.89	70	44.33	20.54				
	3	70	45.97	20.73	73	40.77	19.43	−6.63	2.28	0.004	−0.33
	6	68	44.93	22.33	65	36.97	21.81	−8.98	2.59	0.001	−0.44
	9	69	45.86	20.86	65	36.09	22.23	−10.40	2.81	0.000	−0.51
INQoL Body Image	0	75	61.59	19.83	73	62.77	20.38				
	3	74	61.11	22.20	72	53.96	22.69	−7.89	2.36	0.001	−0.40
	6	70	57.30	23.26	66	44.26	22.78	−12.64	2.88	0.000	−0.63
	9	72	56.40	22.90	66	39.76	25.67	−16.63	3.24	0.000	−0.84
INQoL Emotions	0	75	59.65	24.94	73	66.04	22.49				
	3	74	60.64	22.95	72	58.11	23.59	−6.85	2.60	0.008	−0.29
	6	71	59.41	23.88	66	55.26	23.25	−7.31	2.67	0.006	−0.31
	9	72	59.63	23.15	66	50.58	25.72	−12.72	2.98	0.000	−0.53
INQoL Weakness	0	74	74.66	20.66	73	74.26	20.58				
	3	74	72.46	22.93	73	67.99	20.55	−4.26	2.24	0.057	−0.20
	6	71	72.93	21.47	66	66.77	20.46	−4.97	2.35	0.035	−0.24
	9	72	74.93	22.03	66	66.97	21.66	−6.74	2.54	0.008	−0.32
INQoL Pain	0	75	46.64	28.47	73	44.18	31.03				
	3	74	44.89	28.72	73	36.45	30.51	−6.87	3.08	0.025	−0.23
	6	71	46.07	28.32	66	37.20	30.02	−5.67	3.10	0.068	−0.19
	9	72	44.93	30.27	66	34.14	30.29	−8.76	3.25	0.007	−0.30
INQoL Tiredness	0	75	55.36	27.64	73	60.82	27.03				
	3	74	57.80	25.46	73	56.52	23.69	−4.75	2.97	0.110	−0.18
	6	71	60.03	24.44	66	51.21	27.55	−10.59	3.19	0.001	−0.39
	9	72	60.49	23.95	66	52.23	27.75	−10.79	3.54	0.002	−0.40
HAQ Disability	0	75	1.84	0.82	72	1.72	0.72				
	3	73	1.80	0.83	72	1.70	0.69	0.02	0.04	0.574	0.03
	6	69	1.88	0.79	64	1.70	0.68	−0.05	0.04	0.243	−0.06
	9	72	1.88	0.81	64	1.72	0.72	−0.04	0.04	0.325	−0.06
Work & Social Adjustment	0	75	38.20	15.38	73	35.52	15.64				
	3	73	35.90	16.04	72	31.83	14.38	−1.92	1.50	0.200	−0.13
	6	68	35.52	15.26	64	30.66	15.02	−2.50	1.45	0.084	−0.16
	9	70	36.17	15.51	64	30.46	16.04	−3.69	1.52	0.015	−0.24
HADS Depression	0	75	8.93	3.13	73	9.99	3.08				
	3	74	8.83	3.05	73	8.71	3.38	−0.94	0.38	0.014	−0.31
	6	70	8.55	2.85	66	7.70	3.32	−1.60	0.39	0.000	−0.53
	9	72	8.68	3.17	66	7.71	3.93	−1.80	0.47	0.000	−0.59
HADS Anxiety	0	75	9.48	3.39	73	9.93	3.95				
	3	74	9.59	3.34	73	9.04	3.94	−0.86	0.43	0.045	−0.24
	6	70	9.10	3.32	66	7.08	3.59	−2.30	0.46	0.000	−0.64
	9	72	8.62	3.33	66	6.98	4.43	−2.14	0.56	0.000	−0.60
Acceptance & Action Questionnaire	0	74	27.72	11.73	73	28.50	12.74				
	3	73	26.24	11.99	72	27.60	11.83	0.96	1.23	0.432	0.08
	6	69	26.70	11.96	64	24.09	10.77	−2.97	1.35	0.027	−0.25
	9	71	25.84	10.87	64	23.40	12.62	−2.69	1.52	0.077	−0.23
Mindful Attention Awareness Scale	0	75	4.33	0.82	73	4.08	0.98				
	3	73	4.24	1.00	72	4.14	1.04	0.14	0.11	0.184	0.16
	6	69	4.41	0.89	64	4.33	1.05	0.16	0.10	0.112	0.18
	9	71	4.46	0.93	64	4.44	1.15	0.22	0.12	0.068	0.24
Committed Action Questionnaire	0	75	28.41	7.79	73	28.17	7.70				
	3	72	28.19	8.17	72	29.73	6.83	1.55	0.82	0.058	0.22
	6	68	29.09	7.94	63	30.75	7.53	2.24	0.96	0.020	0.31
	9	71	29.43	8.87	64	31.47	7.82	2.50	1.13	0.027	0.35
Intervention benefit	0	0			0						
	3	65	1.49	1.31	68	2.96	1.41	1.37	0.23	0.000	0.70
	6	56	1.05	1.28	66	3.53	1.54	2.37	0.24	0.000	1.20
	9	60	1.03	1.40	63	3.51	1.65	2.39	0.26	0.000	1.21
Intervention harm	0	0			0						
	3	67	0.51	0.89	71	0.67	0.89	0.11	0.15	0.465	0.11
	6	60	0.44	0.79	66	0.54	0.85	0.06	0.15	0.692	0.06
	9	60	0.64	1.05	66	0.54	0.90	−0.14	0.17	0.411	−0.15
IBM Functional Rating Scale	0	21	22.08	7.76	13	24.46	7.25				
	3	19	21.26	7.53	15	22.80	6.64	−0.67	0.73	.	−0.09
	6	19	20.79	7.90	13	22.38	7.01	−1.47	0.81	.	−0.20
	9	20	21.35	8.16	13	23.38	6.36	−0.99	0.88	.	−0.13

### Therapy integrity

In total 100 sessions were coded by at least one of the two raters. ACTMuS specific Ratings for consistency with ACT were high with mean scores around 6 out of a possible 7, with high levels of agreement between raters (AC = 0.83). Ratings for the therapeutic alliance were high with mean scores around 6 out of a possible 7 and high inter-rater agreement (AC = 0.87). Assessment of fidelity to ACT principles, as measured by the ACT-FM (O'Neill et al., 2019), showed high levels of ACT-consistent therapist behaviours (Session 2, *M* = 23.18, s.d. = 5.69; Session 3, *M* = 21.83, s.d. = 6.78, *ICC* = 0.49).

Adverse event rates were similar across both groups. For the intention to treat the sample, 27 (47.4%) people in the control group reported an adverse event during follow up compared to 30 (52.6%) in the intervention group (Odds ratio = 1.21; 95%CI 0.63–2.34; *p* = 0.565). Four of the events were considered serious: one death in the control group, one non-fatal overdose in the intervention group, one reported incident of suicidal ideation in the intervention group, and one period of breathlessness requiring hospitalisation in the intervention group. None were considered by the ethics committee to be related to participation in the intervention or the trial.

## Discussion

Guided self-help ACT plus SMC was more efficacious than SMC alone, with statistically significant improvements on our primary outcome, QoL, as well as secondary outcomes including mood. As expected from a psychological therapy, measures of muscle disease severity namely the HAQ and the IBM FRS did not change during the trial periods but the impact of these symptoms on functioning (as captured by INQoL Activities subscore and the WSAS) did improve. The pattern of change indicated that change increased over time. Effect sizes were moderate, but particularly encouraging given the brief nature of the intervention. The fact that QoL and levels of distress improved in people with MD, which come with a high symptom burden and few direct treatments for symptoms, is heartening. The results suggest that psychological intervention has applicability in MD beyond being an adjunct treatment for severe fatigue, which has been demonstrated in earlier trials (Okkersen et al., 2018; Voet et al., 2014). Importantly, given that the rationale for a guided self-help approach was to enhance acceptability, there were very few treatment drop-outs and adverse events were minimal. The trial also extends the literature base supporting ACT for improving QoL and distress in chronic diseases (Graham et al., 2016b).

In addition to the more conventional clinical outcomes, some components of psychological flexibility – the psychological process that ACT aims to enhance in order to improve QoL (Hayes et al., 2006) – showed significant improvements favouring the intervention. This included acceptance and committed action, but not mindfulness, and not at all time points, and these effects were generally small. Meta-analyses across mental and physical health conditions suggest variability in the impact of ACT on these process measures (Gloster et al., 2020). The present findings are commensurate with the brevity of the ACTMus intervention, and with results from trials assessing the efficacy of briefer ACT interventions (Godfrey et al., 2019; Hawkes et al., 2013).

There were strengths to this study. It was a fully powered and carefully conducted randomised controlled trial. Recruitment was not just limited to those attending hospital muscle clinics but also from muscle disease registries and charities – perhaps making it more representative of the muscle disease population as a whole. The outcome measure was a disease-specific measure of QoL, which isolates QoL and the impact of MD symptoms on meaningful functioning – an appropriate treatment outcome for a psychological intervention in MD – from symptom severity, which is better treated by physiotherapy or medical intervention. The four groups of MDs chosen for this study represent a large proportion of those with MD. The results may also generalise to those with other types of chronic progressive MD. While the specific pathophysiologies and symptom constellations differ between MDs, the targets of psychological intervention are likely to be common, for example, psychological adjustment, declines in mobility, emotion regulation in the face of a chronic stressor, and the challenge of condition self-management (de Ridder, Geenen, Kuijer, & van Middendorp, 2008; Graham et al., 2015). Therefore, there is growing interest in offering group psychological interventions to participants with differing chronic diseases [e.g. (Brassington et al., 2016; Depping, Uhlenbusch, Härter, Schramm, & Löwe, 2021)]. An exception may be in cases where MD symptoms affect sight or hearing to an extreme extent or in conditions where cognitive functioning is often affected e.g. myotonic dystrophy. Here psychological therapy materials and delivery may require adaptations for issues with working memory, attention etc. The ACT intervention was brief with minimal therapist input from a clinical psychologist (about 2 h telephone contact for each person who completed treatment). The therapist was trained specifically in the ACT and received supervision from an experienced clinical psychologist to ensure therapy integrity. Independent raters showed high inter-rater reliability and provided evidence of high treatment fidelity. Follow up rates in this study were also very high. In terms of risk of bias to the treatment effect itself we did randomise, ensured allocation concealment, and conducted analyses according to a prespecified analysis plan and blind to group allocation. Thus we feel there is a low risk of bias to the treatment effect estimates.

### Limitations and ideas for future research

Although we recruited widely, it remains possible our sample was not representative of the population with muscle disease. Indeed, most participants were white British. This trial is closer to the definition of an explanatory trial within the PRECIS-2 framework (Loudon et al., 2015). We had a short follow up, necessitated by the nature of funding. To know whether gains were maintained longer term, a follow-up assessment of outcomes at 6 months has been completed as an additional study. To fully comply with CONSORT recommendations (Schulz, Altman, & Moher, 2010) this will be reported separately to this per-protocol paper. We did not assess whether the intervention was cost-effective. The issue of whether the treatment could be delivered by less experienced therapists should be evaluated in future trials. Encouragingly, a recent trial reported the benefits of a brief ACT intervention for people with a rare chronic disease that was delivered via peer support (Depping et al., 2021). Although in common with the other trials of psychological interventions in the area (Okkersen et al., 2018; Voet et al., 2014), the comparator arm (SMC alone) did not control for the effect of attention or placebo response to the intervention. Therefore, future trials may want to disentangle the specific treatment effects from the effects of attention or expectancy. Finally, our intervention is fully deliverable within the remote medical and psychological therapy services that have become usual clinical practice since the onset of the coronavirus disease-2019 (COVID-19) pandemic.

In conclusion, this appropriately powered randomised controlled trial found that a brief self-help form of ACT with minimal input from a therapist was more efficacious than SMC. Additional studies are needed to assess whether this finding is replicable, and whether effects persist in the longer term.

## Supplementary material

For supplementary material accompanying this paper visit https://doi.org/10.1017/S0033291722000083. click here to view supplementary material
