# Supplementary material for: A randomised controlled trial of acceptance and commitment therapy for improving quality of life in people with muscle diseases
Source: Psychol Med. 2022 Feb 23;53(8):3511–24. doi: 10.1017/S0033291722000083 (PMC10277769; doi:10.1017/S0033291722000083)
Supplement: Supplementary file 1 [file S0033291722000083sup001.docx]

**ACTMUS Supplementary Material**

**Baseline data by loss to follow-up**

Table 1 and 2, respectively, present baseline demographics for these either not completing any post-randomisation assessment (and thus not analysed) and those not completing the full 9 weeks of follow up compared to the rest of the sample. Those lost to follow-up tended to have lower educational attainment and were less likely to have had prior experience of any kind of psychological intervention. This is unlikely to have any biasing effect on the treatment effect analysis since the number of people lost to follow up was small with the analysis example excluding <5% patients.

Table 1. Baseline variables for those completing at least one post-randomisation assessment (and included in the intention-to-treat analysis) versus those not completing any post-randomisation assessments

| Factor | Lost after baseline (n=7) | Analysed  (n=148) | p-value |
| --- | --- | --- | --- |
| Age, mean (SD) | 56.0 (18.9) | 52.7 (15.4) | 0.58 |
| Female | 2 (28.6%) | 73 (49.3%) | 0.44 |
| Years since diagnosis, median (IQR) | 10.0 (5.0, 15.0) | 13.5 (6.0, 23.5) | 0.25 |
| MD type: Limb Girdle | 2 (28.6%) | 35 (23.6%) | 0.22 |
| Beckers | 1 (14.3%) | 4 (2.7%) |  |
| FSHD | 2 (28.6%) | 74 (50.0%) |  |
| IBM | 2 (28.6%) | 35 (23.6%) |  |
| Age MD onset, mean (SD) | 40.6 (22.0) | 34.1 (20.3) | 0.41 |
| Other diagnoses? | 5 (83.3%) | 80 (54.8%) | 0.23 |
| White ethnicity | 6 (100.0%) | 133 (89.9%) | 1.00 |
| In paid employment | 0 (0.0%) | 63 (42.6%) | 0.082 |
| Low education | 5 (83.3%) | 55 (37.2%) | 0.034 |
| In a relationship | 2 (33.3%) | 98 (66.2%) | 0.18 |
| Lives alone | 2 (33.3%) | 25 (16.9%) | 0.28 |
| Dependents | 3 (42.9%) | 50 (33.8%) | 0.69 |
| Previous psychological experience | 0 (0.0%) | 70 (47.3%) | 0.017 |
| Previous treatment Anx/Dep | 1 (14.3%) | 73 (49.3%) | 0.12 |
| Possible Anx/Dep | 5 (71.4%) | 123 (83.1%) | 0.35 |
| INQoL Total, baseline, mean (SD) | 56.4 (24.0) | 59.8 (17.4) | 0.67 |
| WSAS, mean (SD) | 40.0 (12.4) | 36.9 (15.5) | 0.63 |
| HADS Anxiety, mean (SD) | 9.2 (4.6) | 9.7 (3.7) | 0.73 |
| HADS Depression, mean (SD) | 9.8 (4.2) | 9.5 (3.1) | 0.77 |
| HAQ Disability, mean (SD) | 1.5 (0.8) | 1.8 (0.8) | 0.38 |

Table 2. Baseline variables for those completing at least one post-randomisation assessment vs not

| Factor | Loss by 9 weeks (n=17) | Completed 9 weeks (n=138) | p-value |
| --- | --- | --- | --- |
| Age, mean (SD) | 52.1 (14.7) | 52.9 (15.6) | 0.84 |
| Female | 7 (41.2%) | 68 (49.3%) | 0.61 |
| Years since diagnosis, median (IQR) | 10.0 (8.0, 14.0) | 14.0 (6.0, 24.0) | 0.16 |
| MD type: Limb Girdle | 5 (29.4%) | 32 (23.2%) | 0.19 |
| Beckers | 2 (11.8%) | 3 (2.2%) |  |
| FSHD | 7 (41.2%) | 69 (50.0%) |  |
| IBM | 3 (17.6%) | 34 (24.6%) |  |
| Age MD Onset, mean (SD) | 35.5 (19.6) | 34.3 (20.5) | 0.81 |
| Other diagnoses? | 11 (68.8%) | 74 (54.4%) | 0.30 |
| White ethnicity | 14 (87.5%) | 125 (90.6%) | 0.66 |
| In paid employment | 4 (25.0%) | 59 (42.8%) | 0.19 |
| Low education | 11 (68.8%) | 49 (35.5%) | 0.014 |
| In a relationship | 10 (62.5%) | 90 (65.2%) | 0.79 |
| Lives alone | 3 (18.8%) | 24 (17.4%) | 1.00 |
| Dependents | 8 (47.1%) | 45 (32.6%) | 0.28 |
| Previous psychological experience | 6 (35.3%) | 64 (46.4%) | 0.45 |
| Previous treatment Anx/Dep | 7 (41.2%) | 67 (48.6%) | 0.62 |
| Possible Anx/Dep | 12 (70.6%) | 116 (84.1%) | 0.18 |
| INQoL Total, baseline, mean (SD) | 61.3 (21.0) | 59.5 (17.2) | 0.71 |
| WSAS, mean (SD) | 35.1 (17.1) | 37.2 (15.2) | 0.61 |
| HADS Anxiety, mean (SD) | 9.8 (4.5) | 9.7 (3.6) | 0.94 |
| HADS Depression, mean (SD) | 10.1 (4.1) | 9.4 (3.1) | 0.38 |
| HAQ Disability, mean (SD) | 1.6 (0.9) | 1.8 (0.8) | 0.24 |

**Primary outcome efficacy sensitivity analysis**

Table 3 indicates that the adjusted group differences favour the intervention group and were significant with moderate to large effect sizes at all three time points irrespective of the method used to handle missing data. The intention to treat analysis sample includes all individuals in the group to which they were randomised where post-randomisation data were available. Sensitivity analysis where missing data were imputed using the baseline observation carried forward approach indicated negligible difference in the interpretation of the treatment effect. Further sensitivity analyses using the per-protocol sample, excluding eight individuals who were assumed not to have received a sufficient dose of the treatment, also indicated no substantive difference in the treatment effect.

**Table 3. Treatment effects on primary outcome by analysis sample**

| Analysis sample | Time | Control | | | Intervention | | | Adjusted mean difference | | | |
| --- | --- | --- | --- | --- | --- | --- | --- | --- | --- | --- | --- |
|  |  | N | Mean | SD | N | Mean | SD | Diff | SE | p | SMD |
| ITT | Baseline | 75 | 58.69 | 16.94 | 73 | 60.92 | 17.91 |  |  |  |  |
|  | 3 weeks | 74 | 60.18 | 19.13 | 72 | 54.19 | 18.29 | -7.81 | 1.64 | 0.000 | -0.45 |
|  | 6 weeks | 70 | 58.43 | 19.16 | 66 | 50.30 | 19.37 | -9.36 | 1.90 | 0.000 | -0.54 |
|  | 9 weeks | 72 | 58.86 | 19.57 | 66 | 47.77 | 21.18 | -12.22 | 2.17 | 0.000 | -0.71 |
| LOCF | Baseline | 78 | 59.08 | 16.94 | 77 | 60.29 | 18.04 | - | - | - | - |
|  | 3 weeks | 78 | 60.55 | 18.90 | 77 | 54.42 | 18.66 | -7.44 | 1.58 | 0.000 | -0.43 |
|  | 6 weeks | 78 | 58.54 | 19.60 | 77 | 51.17 | 19.75 | -8.69 | 1.79 | 0.000 | -0.50 |
|  | 9 weeks | 78 | 58.65 | 20.10 | 77 | 48.90 | 21.29 | -11.11 | 2.02 | 0.000 | -0.64 |
| Per-protocol | Baseline | 75 | 58.69 | 16.94 | 69 | 60.72 | 18.25 | - | - | - | - |
|  | 3 weeks | 74 | 60.18 | 19.13 | 68 | 54.31 | 18.52 | -7.49 | 1.66 | 0.000 | -0.44 |
|  | 6 weeks | 70 | 58.43 | 19.16 | 63 | 50.19 | 19.61 | -9.43 | 1.98 | 0.000 | -0.55 |
|  | 9 weeks | 72 | 58.86 | 19.57 | 63 | 47.08 | 21.23 | -12.89 | 2.22 | 0.000 | -0.75 |

Figure 1 is a sensitivity plot presenting estimates from a pattern-mixture modelling approach indicating that under any plausible missing data mechanism that the group differences remain significant. In this analysis, missing values are imputed using the estimate from an analysis assuming data are missing at random plus a fixed value (delta) in the raw units of the INQoL scale. Where delta is 0 the model produces results equivalent to the main analyses assuming data are missing at random. Where delta is approximately 12 the results are broadly equivalent to the baseline observation carried forward approach.


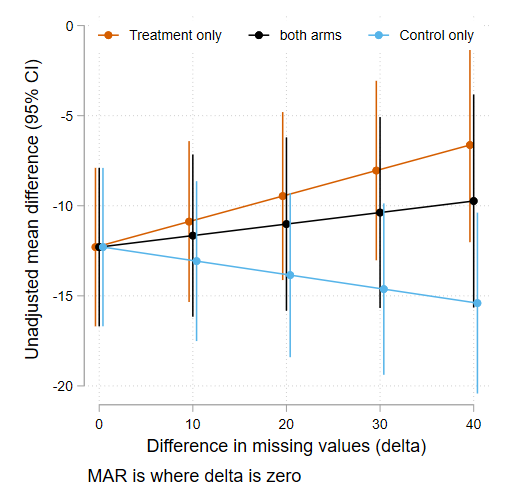


**Figure 1. Pattern-mixture model sensitivity analysis for non-random missingness**
